# Aqueous Humor Proteomic Alterations Associated with Visual Field Index Parameters in Glaucoma Patients: A Pilot Study

**DOI:** 10.3390/jcm10061180

**Published:** 2021-03-12

**Authors:** Sai Karthik Kodeboyina, Tae Jin Lee, Kathryn Bollinger, Lane Ulrich, David Bogorad, Amy Estes, Wenbo Zhi, Shruti Sharma, Ashok Sharma

**Affiliations:** 1 Center for Biotechnology and Genomic Medicine, Medical College of Georgia, Augusta University, Augusta, GA 30912, USA; skodeboyina@augusta.edu (S.K.K.); talee@augusta.edu (T.J.L.); wzhi@augusta.edu (W.Z.); shsharma@augusta.edu (S.S.); 2 Department of Ophthalmology, Medical College of Georgia, Augusta University, Augusta, GA 30912, USA; kbollinger@augusta.edu (K.B.); lulrich@augusta.edu (L.U.); dbogorad@augusta.edu (D.B.); aestes@augusta.edu (A.E.); 3 Department of Population Health Sciences, Medical College of Georgia, Augusta University, Augusta, GA 30912, USA

**Keywords:** aqueous humor, glaucoma, mass spectrometry, proteomics, visual field

## Abstract

Purpose: The purpose of this study was to discover the aqueous humor proteomic changes associated with visual field indices in glaucoma patients. Methods: Aqueous humor samples were analyzed using Liquid Chromatography Tandem Mass Spectrometry (LC-MS/MS). The visual fields were analyzed with the Humphrey Visual Field analyzer. Statistical analyses were performed to discover the relationship between the aqueous humor proteins and visual field parameters including Pattern Standard Deviation (PSD), Visual Field Index (VFI), Mean Deviation (MD) and Glaucoma Hemifield Test (GHT). Results: In total, 222 proteins were identified in 49 aqueous humor samples. A total of 11, 9, 7, and 6 proteins were significantly correlated with PSD, VFI, MD, and GHT respectively. These proteins include apolipoprotein D, members of complement pathway (C1S, C4A, C4B, C8B, and CD14), and immunoglobulin family (IKHV3-9, IGKV2-28). Conclusion: Several proteins involved in immune responses (immunoglobulins and complement factors) and neurodegeneration (apolipoprotein D) were identified to be associated with abnormal visual field parameters. These findings provide targets for future studies investigating precise molecular mechanisms and new therapies for glaucomatous optic neuropathy.

## 1. Introduction

Glaucoma is a progressive optic neuropathy characterized by retinal ganglion cell death resulting in optic nerve atrophy and corresponding visual field loss [1]. The global prevalence of glaucoma is expected to increase to 118 million people in 2040, making it a major health crisis [2]. Functional glaucomatous loss can be assessed via the global indices of Standard Automated Perimetry (SAP) testing such as the Mean Deviation (MD), Pattern Standard Deviation (PSD), Visual Field Index (VFI), and Glaucoma Hemifield Test (GHT) [3]. The MD represents diffuse losses in the visual field compared to age-matched controls. Conversely, PSD represents focal defects that may underlie disease-specific alterations in the visual field [4]. VFI is a global indicator of vision deterioration reflecting retinal ganglion cell loss with values ranging from 0–100% indicating a perimetrically blind eye to perfect vision. GHT measures the asymmetry between the superior and inferior hemifields, categorized as Outside Normal Limits (ONL) or Within Normal Limits (WNL) [3,4].

Aqueous Humor (AH) is a clear fluid that circulates within the anterior segment of the eye and plays an integral role in many ocular health functions, including nutrient and oxygen supply, removal of metabolic waste, ocular immunity, ocular shape, and refraction [5]. Proteins in AH are vital for the maintenance of anterior segment homeostasis and signaling molecules within the AH are implicated in synthesis, degradation, and modification of the Trabecular Meshwork (TM) Extracellular Matrix (ECM) [6]. The elevated levels of TGFβ2 in the AH of glaucoma patients are associated with ECM stiffening within the trabecular meshwork [7,8]. Moreover, AH facilitates the migration of cytokines that stimulate TM cells [9].

Previous studies have demonstrated alterations in several AH proteins in glaucoma patients [10,11,12,13,14]. However, the association between AH protein levels and functional attributes that estimate disease progression is yet to be explored. This study aimed to evaluate the relationship between the AH proteome and visual field indices in glaucoma patients. The study of this relationship may provide us with a better understanding of how molecular alterations (proteins) are linked to changes in functional attributes (visual field metrics).

## 2. Materials and Methods

### 2.1. Subjects

The aqueous humor was collected from 49 patients diagnosed with Primary Open Angle Glaucoma (POAG) undergoing phacoemulsification or glaucoma incisional surgery at the Medical College of Georgia, Augusta University. Patients do not have other eye conditions that may influence study outcome. During the surgery, a clear corneal incision is made through which the aqueous humor is normally evacuated from the anterior chamber and discarded. However, instead of discarding the aqueous humor, samples were collected in Eppendorf tubes. This method of sample collection at the beginning of the surgery is efficient and poses no significant risk to the patient.

The Institutional Review Board at Augusta University approved the study (IRB #611480) and written informed consents were obtained from all study participants. Chart reviews were conducted to record age, race, gender, smoking history, presence of systemic diseases, and intraocular pressure (IOP) levels for all subjects (Table 1).

### 2.2. AH Sample Preparation

Human aqueous humor samples (60 µL) were lyophilized and then reconstituted into 30 µL 8 M urea (Fisher Chemical, Fair Lawn, NJ, USA) in 50 mM Tris-HCl (pH 8; Amersham Biosciences, Uppsala, Sweden). The reduction of the cysteine residues was performed with 20 mM DTT (Sigma-Aldrich; St-Louis, MO, USA) followed by alkylation with 55 mM of iodoacetamide (Sigma-Aldrich; St-Louis, MO, USA). Urea concentration was reduced to below 1 M by adding 240 µL of 50 mM ammonium bicarbonate buffer (Sigma-Aldrich; St-Louis, MO, USA). Protein concentration was measured using a Bradford assay kit according to the manufacturer’s instructions (Pierce, Rockford, IL, USA) and protein digestion was performed using trypsin (1:20 *w*/*w*, 37 °C overnight).

### 2.3. LC-MS/MS Analysis

Digested AH samples were cleaned using a C18 spin plate (Nest Group, Southborough, MA, USA) followed by column separation using an Ultimate 3000 nano-UPLC system (Thermo Scientific Waltham, MA, USA,) and then analyzed with an Orbitrap Fusion tribrid mass spectrometer (Thermo Scientific, Waltham, MA, USA). Reconstituted peptide mixture (6 μL) was trapped and washed on a Pepmap100 C18 trap (5 μm, 0.3 mm × 5 mm; Thermo Scientific, Waltham, MA, USA) using 2% acetonitrile (Fisher Chemical, Fair Lawn, NJ, USA) in water with 0.1% formic acid (Sigma-Aldrich; St-Louis, MO, USA) for 10 min at 20 μL/min and then separated on a Pepman100 RSLC C18 column (2.0 μm, 75 μm × 150 mm) using a solvent gradient of 2 to 40% acetonitrile with 0.1% formic acid at a flow rate of 300 nL/min over 120 min and a column temperature of 40 °C. Eluted peptides were ionized via nano-electrospray ionization with a source temperature of 275 °C, spray voltage of 2000 V, and then introduced into Orbitrap Fusion mass spectrometer. The following instrument settings were used: Data-dependent acquisition in positive mode; orbitrap MS analyzer for precursor scan at 120,000 FWHM from 300 to 1500 m/z; Ion-trap MS analyzer for MS/MS scan in top speed mode (2-s cycle time); dynamic exclusion settings (repeat count 1, repeat duration 15 s, and exclusion duration 30 s); and fragmentation using Collision-induced Dissociation (CID) with a normalized collision energy of 30%.

### 2.4. Protein Identification and Quantification

The raw MS data were processed using the Proteome Discoverer (v1.4, Thermo Scientific, Waltham, MA, USA) and then submitted for a database search against the manually annotated Uniprot-SwissProt database (20,385 entries) using the SequestHT algorithm. Peptide Spectral Matches (PSMs) were validated using the Perculator PSM validator within the Proteome Discoverer software (v1.4, Thermo Scientific, Waltham, MA, USA). Database search included the following search parameters: Mass tolerances were set at 10 ppm for precursor and 0.6 Da for product ion tolerances; static carbidomethylation (+57.021 Da) for cysteine; and dynamic oxidation (+15.995 Da) for methionine and dynamic phosphorylation (+79.966 Da) for serine, threonine, and tyrosine. Proteins that contained similar peptides that could not be differentiated based on MS/MS analysis alone were grouped based on the Principles of Parsimony. A report comprising the protein identities and PSMs (spectrum counts) for each protein was then exported. The PSM counts serve as a semi-quantitative measure for relative protein expression levels in all samples. A list of 222 proteins identified in AH samples is included in Table S1.

### 2.5. Visual Field Measurements

**Figure 1:**
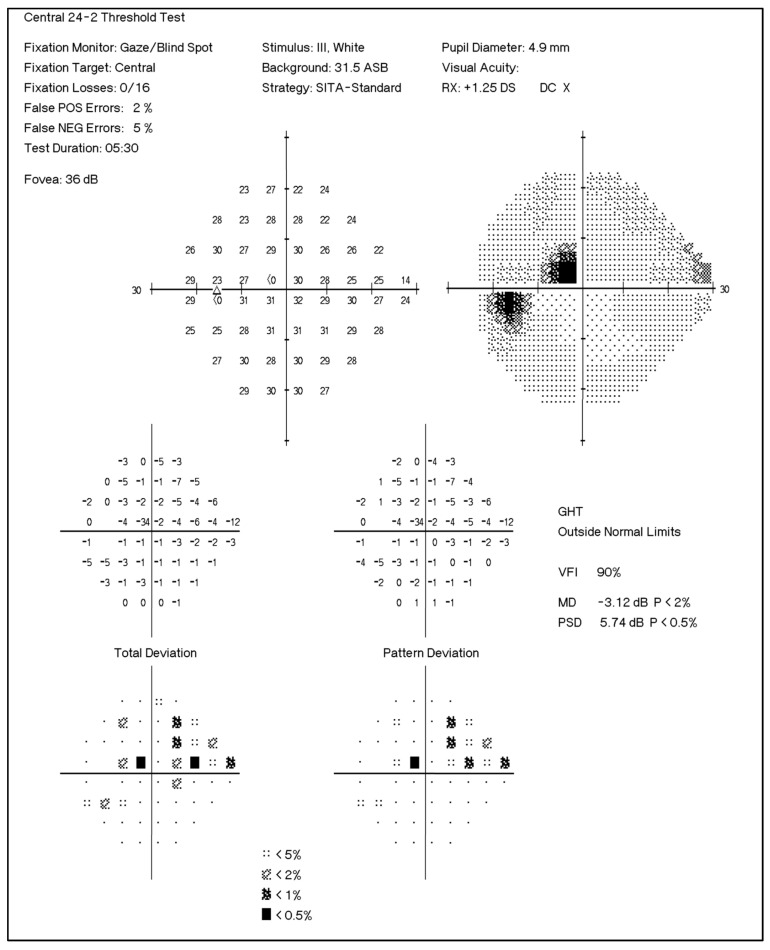
Representative image of the visual field index chart obtained from the Humphrey Visual Field Analyzer II. The test was performed using the standard automated perimetry algorithm with the central 24-2 testing protocol. MD: Mean Deviation, PSD: Pattern Standard Deviation, VFI: Visual Field Index.

The visual fields were assessed with the Humphrey Field Analyzer (Zeiss Humphrey Systems, Dublin, CA, USA), using the Swedish interactive threshold algorithm (SITA) with the appropriate correction for refractive errors. A trained technician performed all visual field testing. During the procedure, patients respond to a series of white light stimuli of varying brightness. The test assesses retina’s sensitivity to detect stimulus at various points within the visual field. The analyzer prints out results with information about measures indicating the status of visual field for the tested eye. MD is obtained from the total deviation plot and indicates overall mean departure from an age-matched normal. All test values at various points on the visual field are added and divided by the number of test locations. Negative value indicates field loss and might be indicative of glaucoma progression. *p* value is assigned if MD is significantly outside normal. PSD is obtained from the pattern deviation plot and highlights focal losses. It reflects the degree of departure of the patient’s visual field pattern from the normal hill of vision. A large PSD reflects an irregular hill of vision and is indicative of glaucoma. GHT assesses five corresponding and mirrored areas in the superior and inferior visual fields. If a significant difference is observed between superior and inferior fields, GHT is categorized as outside the normal limit. A representative visual field chart is shown in Figure 1.

### 2.6. Statistical Analysis

The PSM values obtained from LC-MS/MS analysis were median normalized prior to statistical analysis. Spearman’s correlation analyses were performed between protein levels and PSD, VFI, and MD indices. The Wilcoxon rank sum test was performed to assess proteins differentially expressed between the Outside Normal Limit (ONL) vs. Within Normal Limit (WNL) category of GHT. All statistical analyses were performed using the R Project for Statistical Computing (version 3.6.3, Vienna, Austria).

## 3. Results

### 3.1. Aqueous Humor Proteins Associated with PSD

**Figure 2:**
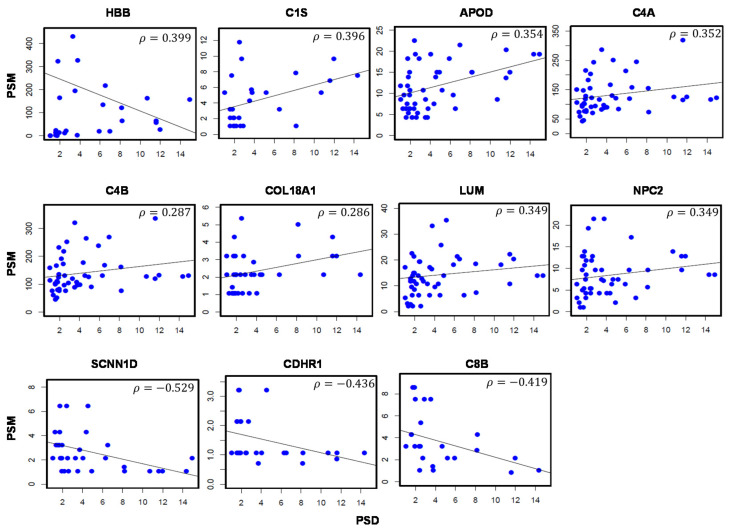
Aqueous humor proteins significantly correlated with Pattern Standard Deviation (PSD) values in glaucoma patients. Scatter plots depict the correlation between protein abundance (PSM; *y*-axis) and PSD measurements (*x*-axis). A total of 11 proteins are significantly correlated with PSD values. *ρ*: Correlation coefficient. *p*-val < 0.05. Sample size (*n*) = 49.

Correlation analysis revealed a total of 11 proteins significantly associated with PSD (Table 2). Plots visualizing the relationship between protein levels and PSD values are shown in Figure 2. Proteins positively correlated with PSD values include the hemoglobin subunit beta (HBB; *ρ* = 0.399), complement C1s subcomponent (C1S; *ρ* = 0.396), apolipoprotein D (APOD; *ρ* = 0.354), complement C4-A (C4A; *ρ* = 0.352), and complement C4-B (C4B; *ρ* = 0.349). Proteins with negative correlations include the amiloride-sensitive sodium channel subunit delta (SCNN1D; *ρ* = −0.529), cadherin-related family member 1 (CDHR1; *ρ* = −0.436), and complement component C8 beta chain (C8B; *ρ* = −0.419).

### 3.2. Aqueous Humor Proteins Associated with VFI

**Figure 3:**
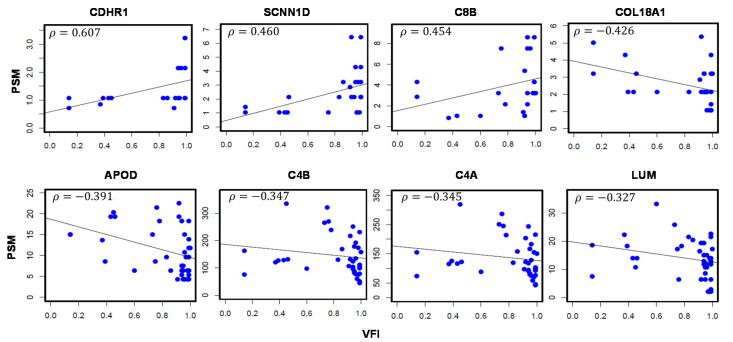
Aqueous humor proteins significantly correlated with Visual Field Index (VFI) values in glaucoma patients. Scatter plots depict the correlation between protein abundance (*y*-axis) and VFI measurements (*x*-axis). Nine proteins are significantly correlated with VFI values. *ρ*: Correlation coefficient. *p*-val < 0.05. Sample size (*n*) = 49.

A total of 9 proteins were associated with VFI, which is a measure of overall visual function (Table 3). Four proteins positively correlated to VFI include: CDHR1 (*ρ* = 0.607), SCNN1D (*ρ* = 0.460), C8B (*ρ* = 0.454), Titin (TTN; *ρ* = 0.399), while 5 proteins with a negative association including Collagen alpha-1(XVIII) chain (COL18A1; *ρ* = −0.426), APOD (*ρ* = −0.391), C4B (*ρ* = −0.347), C4A (*ρ* = −0.345), and Lumican (LUM; *ρ* = −0.327). Correlation plots are presented in Figure 3.

### 3.3. Aqueous Humor Proteins Associated with MD

**Figure 4:**
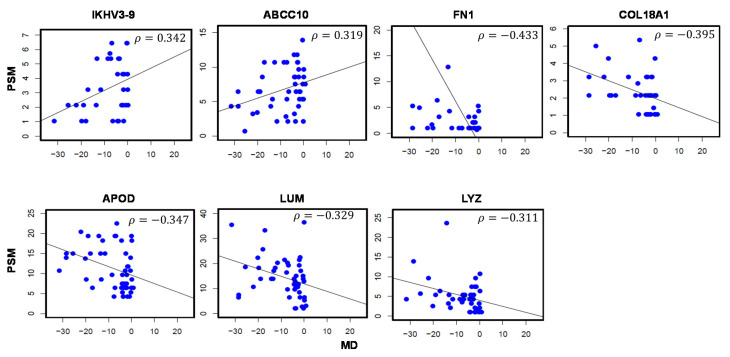
Aqueous humor proteins significantly associated with Mean Deviation (MD) values in glaucoma patients. A total of 7 proteins were significantly correlated with MD values. *ρ*: Correlation coefficient. *p*-val < 0.05. Sample size (*n*) = 49.

MD represents diffuse vision loss and becomes more negative as glaucoma progresses. Two proteins were positively associated and 5 proteins were negatively associated with MD values (Table 4). Correlation plots visualizing the relationship between protein levels and MD values are presented in Figure 4. These 7 proteins include Immunoglobulin heavy variable 3-9 (IGHV3-9; *ρ* = 0.342), multidrug resistance-associated protein 7 (ABCC10; *ρ* = 0.319), fibronectin (FN1; *ρ* = −0.433), COL18A1 (*ρ* = −0.395), APOD (*ρ* = −0.347), LUM (*ρ* = −0.329), and LYZ (*ρ* = −0.311).

### 3.4. Proteomic Changes Associated with GHT

**Figure 5:**
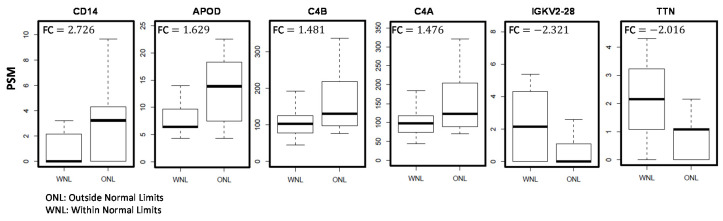
Six proteins associated with deviant glaucoma hemifield (GHT). The subjects were divided into two groups based on the glaucoma hemifield values, Outside Normal Limits (ONL), and Within Normal Limits (WNL). The boxplots depict the distribution of protein levels. Fold Change (FC) represents the alteration in the protein levels in the ONL group as compared to the WNL group. *p*-val < 0.05. Sample size (*n*) = 49.

Analyses were performed to compare the protein levels by dividing the subjects into ONL and WNL groups based on the Glaucoma Hemifield Test (GHT). Four proteins significantly increased with a deviant visual field (ONL) include Monocyte differentiation antigen CD14 (CD14; FC = 2.726), APOD (FC = 1.629), C4B (FC = 1.481), and C4A (FC = 1.476) (Table 5). Two proteins including Immunoglobulin kappa variable 2-28 (IGKV2-28; FC = −2.321) and TTN (FC = −2.061) were decreased in subjects with a deviant visual field. The distribution of protein levels in the two groups is shown in Figure 5.

## 4. Discussion

Functional assessment of the visual field is employed for the diagnosis and staging of glaucoma. Global visual field indices, including MD and PSD represent diffuse and focal sensitivity losses. In this study, we discovered several AH proteins associated with visual field parameters in glaucoma patients. These proteins are involved in several cellular functions, including neurodegeneration, signaling, metabolism, and immune responses.

Glaucoma is classified as a neurodegenerative disease. The role of apolipoproteins in neurodegenerative disorders, particularly Alzheimer’s disease, is well known [15,16,17]. APOD was positively associated with PSD, while negatively associated with VFI and MD values and elevated in the ONL category of GHT. Overall, the differential association of APOD with each of visual field parameters is indicative of its influence on glaucoma progression. In previous studies, mutations and altered levels of apolipoproteins were observed in glaucoma patients [18,19,20].

Earlier studies have shown that immune-related proteins and several members of the complement components including C1S, C1r, C7-C9, and CFH are activated in glaucoma [21,22,23,24]. In this study, we found 7 proteins to be associated with visual field metrics including C1S, C4A, C4B, C8B, IGHV3-9, CD14, and IGKV2-28. C1S, C4A, and C4B were associated with elevated PSD, lower VFI and ONL category of GHT, suggesting their involvement in glaucoma. On the contrary, C8B was positively correlated with VFI and members of the immunolglobulin family such as IKHV3-9 and IGKV2-28 were positively correlated with MD and presented in lower levels in ONL category patients. The complement family of proteins mediate several immune-related processes, including innate, adaptive, and humoral immunity [25,26].

COL18A1 was associated with elevated PSD, and lower VFI and MD reinforcing the importance of collagens in vision disorders. While the role of COL18A1 in POAG is yet to be established, mutation in this protein is associated with angle closure glaucoma [27]. Lumican is a keratin sulfate proteoglycan involved in the maintenance of collagen fibrils, thus influencing corneal transparency. As a key component of extracellular matrix, this protein is involved in maintaining ocular immunologic status [28]. Similar to COL18A1, lumnican positively correlated with PSD and negatively associated with VFI and MD. Lumican has been implicated in aqueous outflow resistance consistent with its over expression in trabecular meshwork of POAG patients compared to normal eyes [29]. CDHR1 belongs to the cadherin superfamily of calcium-dependent cell adhesion molecules that acts as a photoreceptor. This protein negatively correlated with PSD and positively associated with VFI, indicating lower levels as glaucoma progresses. While the relation between the levels of this protein and glaucoma is yet to be explored, mutations in CDHR1 have been associated with retinal dystrophies [30,31].

## 5. Conclusions

In conclusion, this study was a novel attempt to correlate the proteomic changes in aqueous humor with the visual field indices. The proteins discovered in this study may be used as potential targets in future studies to discover novel mechanisms of glaucoma progression.

## Figures and Tables

**Table 1:** Patient demographic characteristics.

Patient Characteristics	Count
POAG patients	49
Female/Male	33/16
AA/Caucasian	29/20
Smoking, N/Y	37/12
Hypertension, N/Y	16/33
Cardiovascular disease, N/Y	46/3
Cerebrovascular disease, N/Y	48/1
Collagen vascular disease, N/Y	43/6
Age (in years)	68.67 ± 11.4
IOP	14.9 ± 9.7

AA: African American, POAG: Primary Open Angle Glaucoma.

**Table 2:** Aqueous humor proteins associated with pattern standard deviation.

UniProt ID	Gene Symbol	Description	Corr.coeff (*ρ*)	*p*-Value
P68871	HBB	Hemoglobin subunit beta	0.399	0.039
P09871	C1S	Complement C1s subcomponent	0.396	0.041
P05090	APOD	Apolipoprotein D	0.354	0.013
P0C0L4	C4A	Complement C4-A	0.352	0.013
P0C0L5	C4B	Complement C4-B	0.349	0.014
P39060	COL18A1	Collagen alpha-1(XVIII) chain	0.349	0.034
P51884	LUM	Lumican	0.287	0.046
P61916	NPC2	NPC intracellular cholesterol transporter 2	0.286	0.046
P51172	SCNN1D	Amiloride-sensitive sodium channel subunit delta	−0.529	0.001
Q96JP9	CDHR1	Cadherin-related family member 1	−0.436	0.026
P07358	C8B	Complement component C8 beta chain	−0.419	0.037

Corr.coeff: Correlation coefficient.

**Table 3:** Aqueous humor proteins associated with Visual Field Index values.

UniProt ID	Gene Symbol	Description	Corr.coeff (*ρ*)	*p*-Value
Q96JP9	CDHR1	Cadherin-related family member 1	0.607	0.002
P51172	SCNN1D	Amiloride-sensitive sodium channel subunit delta	0.460	0.021
P07358	C8B	Complement component C8 beta chain	0.454	0.034
Q8WZ42	TTN	Titin	0.399	0.039
P39060	COL18A1	Collagen alpha-1(XVIII) chain	−0.426	0.021
P05090	APOD	Apolipoprotein D	−0.391	0.014
P0C0L5	C4B	Complement C4-B	−0.347	0.03
P0C0L4	C4A	Complement C4-A	−0.345	0.032
P51884	LUM	Lumican	−0.327	0.042

Corr.coeff: Correlation Coefficient.

**Table 4:** Aqueous humor proteins associated with Mean Deviation (MD).

UniProt ID	Gene Symbol	Description	Corr.coeff (*ρ*)	*p*-Value
P01782	IGHV3-9	Immunoglobulin heavy variable 3-9	0.342	0.048
Q5T3U5	ABCC10	Multidrug resistance-associated protein 7	0.319	0.035
P02751	FN1	Fibronectin	−0.433	0.015
P39060	COL18A1	Collagen alpha-1(XVIII) chain	−0.395	0.016
P05090	APOD	Apolipoprotein D	−0.347	0.016
P51884	LUM	Lumican	−0.329	0.023
P61626	LYZ	Lysozyme C	−0.311	0.048

Corr.coeff: Correlation coefficient.

**Table 5:** Aqueous humor proteins associated with abnormal glaucoma hemifield test.

UniProt ID	Gene Symbol	Description	Fold Change (FC)	*p*-Value
P08571	CD14	Monocyte differentiation antigen CD14	2.726	0.022
P05090	APOD	Apolipoprotein D	1.629	0.011
P0C0L5	C4B	Complement C4-B	1.481	0.046
P0C0L4	C4A	Complement C4-A	1.476	0.046
A0A075B6P5	IGKV2-28	Immunoglobulin kappa variable 2-28	−2.321	0.038
Q8WZ42	TTN	Titin	−2.016	0.028

## Supplementary Materials

The following are available online at https://www.mdpi.com/2077-0383/10/6/1180/s1, Table S1: List of 222 proteins identified in 49 aqueous humor samples.
